# Mapping evidence of depression in HIV-seropositive MSM in sub-Saharan Africa: a scoping review protocol

**DOI:** 10.1186/s13643-021-01604-w

**Published:** 2021-02-05

**Authors:** Delarise M. Mulqueeny, Senzelokuhle M. Nkabini, Manduleli H. Pokiya

**Affiliations:** 1 Department of Social Work, Faculty of Arts, University of Zululand, Private Bag X1001, KwaDlangezwa, 3886 South Africa; 2 Discipline of Public Health Medicine, School of Nursing and Public Health, University of KwaZulu-Natal, Durban, 4001 South Africa; 3 Department of Social Science, Gender and Education, School of Education, University of KwaZulu-Natal, Room 01-032, 121 Marianhill Rd, Pinetown, 3605 South Africa

**Keywords:** Depression, Men who have sex with men, PLHIV, MSM, Sub-Saharan Africa

## Abstract

**Background:**

Depression is one of the most prevalent mental disorders among an estimated 25.6 million people living with HIV (PLHIV) in sub-Saharan Africa (SSA). The depression rate is higher in HIV-seropositive men who have sex with men (MSM) regardless of their sexual orientation, identity or romantic attraction. This is due to various types of stigma including HIV-related stigma, social stigma, self-stigma and mental health stigma. Opportunistic infections, unemployment, poverty and food insecurity also predispose HIV-seropositive MSM to depression. Moreover, depression in heterosexual and sexual minority groups challenges and additionally burdens SSA health care systems due to inadequate economic developments, lack of mental health professionals who specialise in the treatment of depression, few MSM-centred facilities, inadequate mental health infrastructure (hospitals and clinics) and complimentary resources. Although studies have highlighted links between mental health disorder, an HIV diagnosis and sexual minority groups, there is limited research that focusses on depression and its causal factors in MSM living with HIV in SSA. Hence, the relevance of conducting this scoping review.

**Methods:**

A scoping review guided by Arksey and O’Malley’s framework, the enhancements and recommendations of Levac, Colquhoun and O’Brien, Daudt and associates and the 2015 Johanna Briggs Institute’s guidelines will be conducted. Systematic electronic searches of databases and search engines such as Google, Google Scholar, CINAHL (EBSCOhost), MEDLINE (Ovid), and PsycInfo (Ovid) will be conducted to attain published peer-reviewed articles of all study designs. Grey literature will be sourced from media and conference abstracts and reports, governmental reports and unpublished dissertations and theses. Additionally, websites of humanitarian organisations and other relevant departmental websites will also be searched. Literature published between 2010 and 2020 that meets the review’s inclusion criteria, research question and sub-question will be included in this review. All the retrieved literature will be exported to an Endnote X9.2 library after duplicates have been removed.

**Discussion:**

We anticipate mapping relevant literature on depression and the causal factors in HIV-seropositive MSM living in SSA. Once analysed and summarised, the data will be useful in identifying literature gaps, informing systematic reviews and future research. The findings could also assist in depression and sexuality dialogues, and awareness campaigns that address mental health issues, stigma and discrimination among this key population living in SSA.

**Supplementary Information:**

The online version contains supplementary material available at 10.1186/s13643-021-01604-w.

## Background

Sub-Saharan Africa (SSA), like other global regions, records a high prevalence of depression among people living with HIV (PLHIV) [1, 2]. In the context of this review, depression, also known as major depressive disorder (MDD), refers to a mental disorder ‘characterized by low mood, diminished self-worth, pessimistic thoughts, poor concentration, and biological symptoms (that of poor appetite and sleep difficulties) and increased withdrawal from social activities’ [3, 4]. In the context of this review, HIV-seropositive ‘men who have sex with men’ (MSM) refers to men who have sex with men regardless of their sexual orientation, romantic attraction or identity [5]. In 2019, there were 25.6 million PLHIV and 1,080,000 new HIV infections with the rate of depression in SSA being between 9 and 32% [1, 6, 7]. According to The Joint United Nations Programme on HIV/AIDS (*UNAIDS*) MSM are one of the five key populations vulnerable to HIV infection and account for 18% of the estimated 2019 new HIV infections [8].

Depression in HIV-seropositive MSM living in SSA has political, health, societal and economic implications and consequences with romantic sexual acts and relationships between people of the same gender being criminalised in 24 countries in this region [1, 9, 10]. Additionally, less than half (42%) of the 46 countries located in SSA offer resolute constitutional protection and recognise the human rights of their gay and bisexual civilians and practice legal tolerance [10]. Moreover, many healthcare systems and facilities in SSA are compromised and overburdened due to inadequate economic developments, lack of mental health professionals who specialise in the treatment of depression and MSM and lack of specialised mental health infrastructure (hospitals and clinics), MSM services and complimentary resources [11, 12]. Furthermore, only five countries in SSA have functioning mental health programmes that promote the awareness and anti-stigmatisation of mental health issues including depression [13]. Besides, HIV and AIDS national and societal projects and initiatives primarily focussing on MSM are limited or non-existent in some SSA countries due to widespread homophobia [14].

Many MSM experience multiple forms of stigma including sexuality-related stigma, HIV-related stigma, stigma at health facilities, mental health stigma, self, public and social stigma and homophobia, isolation and exclusion [15–17]. These homophobic and transphobic acts and practices as well as the internal and social stigmatisation and marginalisation of MSM, render sexual minorities more susceptible to depression [1, 2, 18]. Moreover, opportunistic infections, food insecurity, unemployment, stress and poverty are also contributory factors predisposing HIV-seropositive MSM to depression [19, 20]. Studies have highlighted that the implications and consequences of challenging healthcare systems, internal and public stigma, homophobia and comorbidities on MSM can lead to suicide attempts, poor quality of life, reduced retention in HIV care, virological failure, isolation, risky behaviour, substance abuse and misuse and non-adherence to antiretroviral treatment (ART) and mortality [21–23].

Furthermore, depression, homophobia and increasing HIV infection rates in key populations including MSM are considered global challenges by the United Nations and are included in the Sustainable Development Goals (SDG’s) [24]. The SDG 3: *Good Health and Well-being*, SDG 5: *Gender Equality*, SDG 10: *Reduced inequality* and SDG 16*: Peace and justice strong institutions* are especially relevant as they promote equality, justice, patient-centred care, community and mental health awareness [25]. Hence, various humanitarian organisations including the World Health Organization (WHO); The US Presidents Emergency Plan for AIDS Relief (PEPFAR); Intersex, Lesbian, Gay, Bisexual and Trans Alliance (ILGA); and the African Commission on Human and People’s Rights (ACHPR) support, promote and enforce programmes that raise awareness on the human rights of all individuals irrespective of their gender and sexual identities and promote inclusive health and education [26, 27]. These organisations also advocate for the eradication of stigma and discrimination associated with mental health, sexual and gender diversity as well as HIV and AIDS.

To avoid duplicating previous reviews and studies conducted on the topic and deciding on whether a systematic or scoping review was more appropriate, two screeners (SMN, MHP) conducted electronic searches of the Cochrane database of systematic reviews and several search engines and databases. These searches revealed that few systematic reviews and several primary studies had been conducted on depression in HIV-seropositive people living in east Africa and SSA and highlighted the relationship between HIV, mental health disorders and sexual minorities with specific emphasis on gay and bisexual men [28–32]. However, to the best of our knowledge, a scoping review on depression and the causal factors thereof among HIV-seropositive MSM living in SSA regardless of their sexual orientation, romantic attraction or identity has not been conducted within the last decade [2, 33]. This is despite the negative consequences on their quality of life and wellbeing. Hence, the results of this scoping review could close that gap by divulging existing evidence including the contributory factors of this disorder to an inclusive MSM population. The objectives framing this review are (a) mapping existing evidence from literature regarding depression in HIV-seropositive MSM in SSA, (b) mapping the various contributory factors that lead to depression in HIV-seropositive MSM in SSA, and (c) identifying gaps in the body of knowledge that could inform future primary studies and systematic reviews.

## Methods

After considering the various information synthesis methods, the team decided a scoping review would be best suited to achieving the study objective of mapping available evidence on depression among HIV-seropositive MSM living in SSA [34]. Additionally, as this is a broad topic, this approach was appropriate to scope studies that used qualitative, quantitative and mixed method research designs including those with descriptive data and to identify key concepts and gaps in knowledge for further research [35]. Moreover, it could assist in assessing the feasibility of conducting systematic reviews in the future whilst avoiding the duplication of previous studies on this topic. Arksey and O’Malley’s framework and enhancements by Levac, Colquhoun and O’Brien, as well as Daudt and associates and the 2015 Johanna Briggs Institute’s guidelines, will guide this review [36–39]. The five stages of the framework are (i) identifying the research question, (ii) identifying relevant studies, (iii) selection of eligible studies, (iv) charting of data and (v) collating, summarising, and reporting the results. To ensure all these steps are followed the Preferred Reporting Items for Systematic Reviews and Meta-Analysis: Extension for Scoping Review guidelines (PRISMA-ScR) will guide the process [40]. Additionally, the results will be presented and summarised using the Preferred Reporting Items for Systematic Review and Meta-Analysis Protocols (PRISMA-P) 2015 checklist to ensure a rigorous process [41–43].

### Identifying the research question

The primary research question guiding this review is: What evidence exists regarding depression in HIV-seropositive MSM in sub-Saharan Africa?

Sub-question:

- What evidence exists regarding the factors that contribute to depression in HIV-seropositive MSM in sub-Saharan Africa?

#### Eligibility criteria

A PCC (*P*opulation, *C*ontext, *C*oncept) framework (Table 1) will adequately address the research question and eligibility of selected and included literature.

**Table 1:** Population, Concept, Context [PCC]

**P** - Population	‘Men who have sex with men living with HIV’ refers to HIV-seropositive males who are 18 years and older and engage in sex with men regardless of their sexual orientation, romantic attraction or identity [5].
**C** - Concepts	Depression: refers to a mental disorder ‘characterized by low mood, diminished self-worth, pessimistic thoughts, poor concentration, and biological symptoms [that of poor appetite and sleep difficulties] and increased withdrawal from social activities’ [4]. The causes of depression include stigma due to sexual orientation, HIV diagnosis and low self-esteem, chemical imbalances, stress, treatment and isolation [2, 15–23].
**C** - Context	Sub-Saharan Africa: Includes 46 countries that are geographically located south of the Saharan desert [44]. Sub-Saharan Africa comprises of 46 of Africa’s 54 countries and excludes Algeria, Djibouti, Egypt, Libya, Morocco, Somalia, Sudan and Tunisia.
**Sources of evidence**	Grey and empirical literature containing evidence of depression and its causal factors in HIV-seropositive MSM living in sub-Saharan Africa.
**Publication Year range**: 2010 and 2020	**Language:** All

### Identifying relevant studies

Qualitative, quantitative and mixed methods studies including descriptive data on depression and the causal factors of depression will be included in the review with the inclusion criteria, research question and sub-question forming the basis for all included literature. Empirical and peer-reviewed literature will be sourced from searches of electronic databases and search engines such as Google, Google Scholar, EBSCOhost, CINAHL, MEDLINE, PsycInfo, World Health Organization (WHO) and education departments and institutions of higher learning websites. Whilst, grey literature (conference abstracts, presentations and reports, government publications including white papers and working papers, unpublished dissertations and theses, policies) will be sourced from humanitarian organisations and relevant departmental and conference websites [45]. Additional searches will include screening citations in reference lists of articles relevant to the topic. A subject librarian, well versed in scoping reviews, will also assist with the sourcing of relevant literature. The research assistant will contact the corresponding authors to attain literature that is electronically unavailable. A draft search strategy using Boolean terms ‘AND’ and ‘OR’ to separate search words and terms will be piloted. Additional file 2 will contain the draft search strategy with keywords, search string, Boolean terms, databases and the number of articles retrieved. The pilot search will be restricted to humans with no language restrictions and a search timeline between 2010 to 2020. Review and research articles are the limiters.

### Selection of eligible studies

The protocol and review team will comprise of three screeners and a research assistant. The research assistant will ensure all retrieved literature is exported to an Endnote X9.2 library. Duplicate documents will be deleted prior to the commencement of title and abstract screening. The electronic library will be shared with all three screeners. Two screeners (DMM, MHP) will independently conduct title and abstract screening with the third screener (SMN) resolving any discrepancies or conflict. Thereafter two screeners (DMM, SMN) will independently conduct full article screening with MHP resolving any conflict and discrepancies that arise. All literature deleted from the Endnote library will be saved in a separate folder to facilitate the study being reproduced.

#### Inclusion criteria

The following criteria will ensure the inclusion of:

- Literature relating to depression and its causal factors among HIV-seropositive MSM in sub-Saharan Africa.
- Literature reporting on incidents of depression among HIV-seropositive MSM in sub-Saharan Africa.
- Studies with descriptive data on depression.
- Grey literature (government reports, policy statements, conference proceedings, theses and dissertations) relating to depression among HIV-seropositive MSM in sub-Saharan Africa.
- Articles published between 2010 and 2020 as a larger timeframe will adequately capture this understudied topic.
- Studies conducted in all languages.

#### Exclusion criteria

- Literature that does not include content focusing on depression and the causal factors of depression among HIV-seropositive MSM in sub-Saharan Africa.
- Literature published prior to 2010.
- Articles focussing on female HIV-seropositive individuals.

**Fig. 1:**
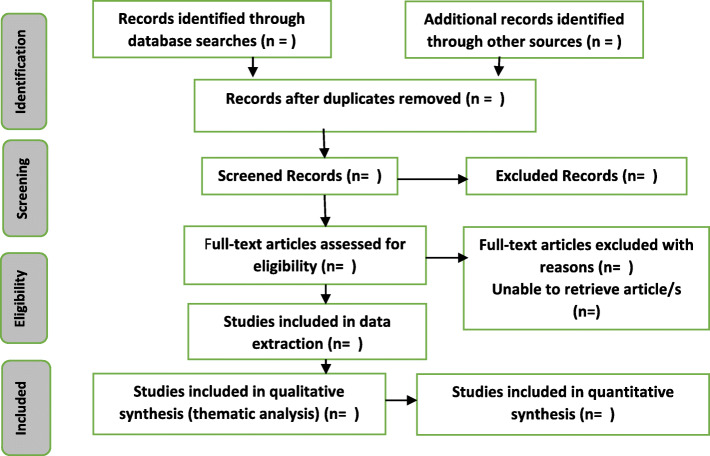
PRISMA ScR flowchart demonstrates the literature search and study selection processes

A summary of the inclusion and exclusion screening process will be evident in the PRISMA ScR flowchart (Fig. 1).

### Charting of data

A data charting table (Table 2) was created in a Google form and will be populated with literature that possesses variables or themes that comprehensively answer the research question. Two screeners (DMM, MHP) will independently electronically populate the table. Discrepancies will be resolved by SMN in collaboration between MHP and DMM. This iterative process necessitates that the data charting table is regularly updated to ensure accuracy, it being current and ensuring a rigorous process is followed.

**Table 2:** Data charting table

Author and year of publication	
Article title	
Study aim	
Country	
Study design/methodology	
Study setting	
Study population	
Duration of the study	
Factors contributing to depression	
Population age	
Relevant/significant findings	
Key conclusions of article	
Notes	

### Collating, summarising and reporting the results

As this scoping review will map quantitative, qualitative and mixed method literature relating to depression among HIV-seropositive MSM in SSA, a three-pronged approach will be used by all three screeners during this stage. The stages are (1) thematic content analysis for qualitative studies and numerical counts and tables for quantitative studies, (2) narrative account for summarising and reporting all the results and (3) identification and summarising literature gaps, feasibility of conducting systematic reviews or further primary studies and where relevant the implications for policy and practice interventions. The use of the NVivo data analysis software and Braun and Clarke’s thematic framework will guide the qualitative analysis and the Microsoft Excel programme will be used for the quantitative analysis [46]. This process will incorporate the minority stress theory (external prejudice including distal stressors and syndemic theory conceptual framework to synthesise the contributory factors of depression in MSM irrespective of their sexual attraction or identity) [47]. To avoid any bias and discrepancies, reflexive meetings will be held to resolve any conflict or disagreements by consensus throughout the process [48].

#### Synthesis/quality appraisal

To avoid and report on any risk of bias and ensure that included evidence is appropriate, this review will utilise the mixed-method appraisal tool (MMAT) version 2018 to appraise the quality of all included evidence [49]. Two screeners (DMM, MHP) will be responsible for assigning ratings of 100% for high average articles, 75% for above average articles, 50% average and 25% for low-quality articles.

#### Discrepancies between the protocol and the scoping review

Any discrepancies between the protocol, the actual review and the reasons and consequences thereof will be reported in the final report.

## Discussion

Depression experienced by HIV-seropositive MSM has physical, educational, social, financial, psychological and health short- and long-term consequences which could further burden SSA health organisations and systems [50]. This mental disorder can manifest due to the shock and unwillingness to accept the HIV diagnosis, deciding on whether to disclose, nondisclosure of the prognosis, lack of social support and the hesitation and commencement or rejection of antiretroviral treatment (ART) [30, 51]. Additionally, untreated depression in HIV-seropositive MSM can lead to risky sexual behaviour, alcohol and drug misuse and abuse and suicide [52]. Moreover, mental illness accounts for approximately 800,000 suicide deaths every year thus highlighting the relevance of this review being conducted [53].

The strengths of conducting this review are mapping evidence of an under-researched topic on a key population in SSA. Hence, the results emanating from this scoping review could emphasise the relationships between an HIV diagnosis, mental health disorders such as depression and the need for further investigation and attention. Such findings could be useful to humanitarian, mental health, governmental and healthcare organisations and address and direct the role of governments, community-based organisations (CBOs), non-governmental organisations (NGOs) and policies and awareness campaigns. Additionally, it could contribute to mental health, sexuality and chronic illness dialogues that address depression, stigma, patient-centred care, gender identity and discrimination and inform the development of patient-centred mental health programmes and interventions for key populations within SSA.

This study could assist in mapping out literature gaps pertaining to depression in HIV-seropositive MSM living in SSA and offer suggestions on how further research can address and close these gaps. The results of the proposed study could help to support advocacy activities that aim to integrate psychological consultation and counselling activities in HIV services for key populations and mental health. The outcomes of this scoping review would be published in peer-reviewed journals, government reports and policy statements and presented at international and national seminars.

## Limitations

Having no language restriction could be a costly and lengthy process. However, assigning timelines to each stage of the review could ensure deadlines are adhered to and the process is completed in the assigned time.

## Supplementary Information

**Additional file 1.** PRISMA-P 2015 Checklist.

**Additional file 2: Table 2**. Draft search for MEDLINE/EBSCOhost.

## Data Availability

All data generated or analysed during this study will be included in the published scoping review article and will be available upon request.

## Abbreviations

ACHPR: African Commission on Human and People’s Right
ART: Antiretroviral treatment
CBOs: Community-based organisations
HIV: Human immunodeficiency virus
HRW: Human Rights Watch
ILGA: International Lesbian, Gay, Bisexual, Trans and Intersex Association
MDD: Major depressive disorder
MSM: Men who have sex with men
NGO: Non-governmental organisations
PEPFAR: The US Presidents Emergency Plan for Aids Relief
PLHIV: People living with HIV
SDG: Sustainable Development Goal
SSA: Sub-Saharan Africa
UNAIDS: The Joint United Nations Programme on HIV/AIDS
WHO: World Health Organization
