# Supplementary material for: Mapping evidence of depression in HIV-seropositive MSM in sub-Saharan Africa: a scoping review protocol
Source: Syst Rev. 2021 Feb 5;10:50. doi: 10.1186/s13643-021-01604-w (PMC7866456; doi:10.1186/s13643-021-01604-w)
Supplement: Supplementary file 2 — Additional file 2: Table 2. Draft search for MEDLINE/EBSCOhost. [file 13643_2021_1604_MOESM2_ESM.docx]

**Table 2: Draft search strategy**

| Keywords | Search  Date | Search engine | Results |
| --- | --- | --- | --- |
| Depression OR MDD OR major depressive disorder*,* AND Men who have Sex with Men OR bisexual men young people OR young adults OR young males OR males AND gay males OR gay men AND sub-Saharan Africa OR south of the Saharan desert |  | EBSCOhost |  |
| [[[[[[["depressive disorder" [MeSH Terms] OR  ["depressive" [All Fields] AND  "disorder"[All Fields]] OR "depressive disorder"[All Fields] OR "depression"[All Fields] OR "depression" [MeSH Terms]] AND  "young"[All Fields]] AND  ["young adult"[MeSH Terms] OR ["young"  [All Fields] AND  "adult"[All Fields]] OR "young adult" [All Fields] OR ["young"[All Fields] AND  "adults" [All Fields]] OR "young adults"[All Fields]]] AND ["young"[All Fields] AND  ["persons" [MeSH Terms] OR "persons"[All Fields]  OR "people"[All Fields]]]] AND  ["HIV seropositivity"[MeSH Terms] OR ["HIV"[All Fields] AND  "seropositivity" [All Fields]] OR "hiv seropositivity"[All Fields] OR ["HIV"[All Fields] AND "positive"[All Fields]] OR "HIV positive"[All Fields]]]] OR “HIV infected” AND  [["sexual and gender minorities"[MeSH Terms] OR ["sexual"[All Fields] AND  "gender"[All Fields] AND  "minorities"[All Fields]] OR "sexual and gender minorities"[All Fields] OR "gay"[All Fields] OR "homosexuality"[MeSH Terms] OR "homosexuality"[All Fields]] AND  ["men"[MeSH Terms] OR "men"[All Fields]]]] AND ["Africa south of the Sahara" [MeSH Terms] OR ["Africa"[All Fields] AND  "south"[All Fields] AND  "Sahara"[All Fields]] OR  "Africa South of the Sahara"[All Fields] OR ["sub"[All Fields] AND "saharan"[All Fields] AND "Africa"[All Fields]] OR "sub-Saharan Africa"[All Fields]] AND  ["2010/01/01"[PubDate]: "2020/01/31"[PubDate]] |  | MEDLINE |  |
